# A Zinc Chelate of Lysine/Glutamic Acid Reduces Alcohol-Induced Ciliary Dysfunction

**DOI:** 10.3390/biology15151222

**Published:** 2026-07-23

**Authors:** Emily Do, Daren L. Knoell, Christopher D. Bauer, Deanna D. Mosley, Deandra R. Smith, Derrick R. Samuelson, Todd A. Wyatt

**Affiliations:** 1 Department of Internal Medicine, Pulmonary, Critical Care and Sleep Medicine Division, University of Nebraska Medical Center, 985910 Nebraska Medical Center, Omaha, NE 68198-5910, USA; edopharmd@hotmail.com (E.D.); daren.knoell@unmc.edu (D.L.K.); christopher.bauer@unmc.edu (C.D.B.); deanna.mosley@unmc.edu (D.D.M.); derrick.samuelson@unmc.edu (D.R.S.); 2 Department of Environmental, Agricultural, and Occupational Health, University of Nebraska Medical Center, 984388 Nebraska Medical Center, Omaha, NE 68198-4388, USA; 3 Department of Pharmacy Practice and Science, University of Nebraska Medical Center, 986120 Nebraska Medical Center, Omaha, NE 68198-6120, USA; deandra.smith@unmc.edu; 4 VA Nebraska-Western Iowa Health Care System, 4101 Woolworth Avenue, Omaha, NE 68105-1850, USA

**Keywords:** lung, alcohol, cilia, zinc, protein kinase, phosphatase

## Abstract

Alcohol misuse is a known risk factor for pneumonia, and up to 50% of individuals who misuse alcohol are zinc deficient. Chronic alcohol misuse can lead to alcohol-induced ciliary dysfunction (AICD), preventing effective mucociliary clearance and protection against lung infection. Because zinc salts fail to alter AICD, we hypothesized a form of zinc (Zinpro Zinc LG) with enhanced cell uptake would protect against AICD. Human and mouse airway epithelial cells were treated with 50 mM ethanol for 1–24 h followed by the ciliostimulatory agents. Cilia beat frequency (CBF) and cAMP-dependent protein kinase (PKA) activity were assayed. Cyclic AMP or beta agonists stimulated a significant increase in CBF, but 24 h alcohol pretreatment resulted in the desensitization of the cilia to these agents. Pretreatment of cells with 1–10 µg/mL Zinpro Zinc LG prior to alcohol restored the cAMP cilia stimulation response. Zinpro Zinc LG alone produced no effects and was not toxic at <10 µg/mL. PKA activation was prevented in cells treated with alcohol for 24 h, but pretreatment with Zinpro Zinc LG prior to alcohol restored kinase activation. Zinpro Zinc LG prevented alcohol-stimulation of Protein Phosphatase-1, the regulator of PKA desensitization. These data demonstrate that the exposomal combination of alcohol and nutritional Zn deficiency could lead to an aberrant ciliary clearance response that may be prevented by effective zinc supplementation.

## Simple Summary

Evidence of human consumption of alcohol predates recorded history. However, excessive use of alcohol is associated with many chronic diseases and negative health effects. For over 250 years, the scientific literature has documented an increase in lung infections in those individuals who misuse alcohol. While there are many possible causes of these increased infections, one known injury from alcohol is to the cilia of the lung airways. Cilia are hair-like extensions of the cells that line the airways, which are necessary to propel inhaled particles, viruses, and bacteria out of the lung by increasing their movement in a coordinated fashion. Alcohol exposure prevents this increased movement. Because people who misuse alcohol are at increased risk for zinc deficiency and zinc supplementation is known to reduce the duration and severity of some lung infections, we investigated whether a form of zinc with enhanced absorption qualities would be protective against alcohol-damaged cilia. Our results identified the mechanism for zinc-mediated preservation of ciliary function against this alcohol-induced injury and identified a potential use for zinc in minimizing the public health burden of alcohol-associated lung infections.

## 1. Introduction

The vast majority (85%) of adults in the US have consumed or do consume alcohol. Unfortunately, this is not without consequences, as, according to the National Survey on Drug Use and Health, about 22% of those have engaged in episodic binge drinking, and approximately 28 million people (10%) in the US had an identified alcohol use disorder (AUD) in 2024 [1]. While behavioral and social consequences of alcohol misuse result in significant emotional and physical trauma as well as economic costs, heavy and prolonged alcohol consumption leads to multiple forms of target organ tissue injury, including hepatic, pancreatic, intestinal, cardiovascular, neurological, endocrine, musculoskeletal, hematological, and pulmonary damage [2].

Because the lung is a target organ for alcohol effects, individuals with AUD are more likely to develop respiratory diseases such as pneumonia, tuberculosis, respiratory syncytial virus infection, and acute respiratory distress syndrome [3,4]. The increased susceptibility for these infections and other pulmonary complications is due to the damaging effects of alcohol on the immune system [5]. Previous studies have observed that prolonged alcohol use can alter pro-inflammatory cytokine release, which can also promote worsening severity of an infection [6]. Chronic alcohol ingestion causes impaired pathogen clearance through desensitization of cilia caused by alcohol activation of protein phosphatase 1 (PP1), leading to the desensitization of the cAMP-dependent protein kinase (PKA) [7] and inability for bacterial phagocytosis through production of oxidative stress in the alveolar space [8]. Lung injury is compounded by the estimation that as many as 80% of individuals with AUD are cigarette smokers [9]. However, the impact of alcohol misuse on the lungs is multifaceted, and the alcohol exposome [10], consisting of environmental and occupational exposures or nutritional deficiencies, is less defined.

Indeed, nutrient deficiencies are common among those with AUD, as alcohol calories displace food nutrients, leading to alcohol-induced impairment of nutrient absorption and metabolism due to the increase in alcohol-metabolization toxins as well as the loss of vitamins and minerals [11]. A key element impacted by reduced absorption and increased excretion in response to alcohol consumption is zinc [12,13] and zinc deficiency can lead to several known dysregulated lung functions [14,15,16]. With regard to mucociliary clearance, zinc has been shown to stimulate nasal cilia beating [17], but zinc cellular uptake and subcellular distribution of elemental zinc in mammals is restricted to entry via two highly conserved families of proteins: the Zinc Transporter (ZnT) and Zrt, Irt-like (ZIP) transporters [18]. Recently, we have found that ciliated cell supplementation with amino acid conjugates of zinc could reverse organic dust-induced cilia slowing [6].

Therefore, we hypothesized that Zinpro Zinc LG, a zinc chelate of lysine and glutamic acid, which has enhanced cellular uptake, would provide protection against alcohol-induced ciliary dysfunction in airway epithelial cells. To test this hypothesis, we used both mouse and human airway cells exposed to alcohol and examined the role of Zinpro Zinc LG in reversing cilia desensitization. We also investigated the impact of this zinc supplementation on the alcohol-mediated regulation of cilia kinases and phosphatase activity that governs motility. Effective zinc supplementation may prove to be one approach in the prevention of alcohol-mediated lung infections.

## 2. Materials and Methods

### 2.1. Cell Culture

Human and mouse airway epithelial cells were used for this study. BEAS-2Bs are a non-tumorigenic, human bronchial epithelial cell line, which were obtained from the American Type Culture Collection (ATCC, Manassas, VA, USA). The transformed human bronchial epithelial cell line, 16HBE14o- [19], was originally purchased from Dr. D. C. Gruenert of the Cardiovascular Research Institute of the University of California, San Francisco. The 16HBE14o- cells were grown in Dulbecco’s Modified Eagle’s Media (DMEM; Invitrogen-Thermo Fisher, Waltham, MA, USA) supplemented with 10% fetal bovine serum (FBS) and penicillin/streptomycin. BEAS-2B and 16HBE cultures were grown at 37 °C with 5% CO_2_ in M-199 media or Dulbecco’s modified Eagle’s Media, respectively. Cells were passaged by adding Gibco TrypLE Express (Gibco-Thermo Fisher, Grand Island, NY, USA) for 5–10 min and TrypLE Express inactivated with phosphate-buffered saline (PBS; pH 7.4) containing 10% fetal bovine serum (FBS) (R&D Systems-BioTechne, Minneapolis, MN, USA). Collected cells were centrifuged for 10 min at 193× *g* (Beckman Coulter, Brea, CA, USA), and an additional wash was performed with PBS containing 10% fetal bovine serum (FBS). Cells were counted, plated, and allowed to proliferate to 80–90% confluence for subsequent assays.

Wild-type 8-week-old male C57BL/6 mice (Jackson Laboratory, Bar Harbor, ME, USA) were euthanized, and the tracheas were removed and cut longitudinally to expose the inner lumen. The tracheas were incubated overnight at 4 °C in 1.5 mg/mL Pronase digestion buffer (Sigma-Aldrich, St. Louis, MO, USA). Digestion was halted with 10% FBS, tracheas removed, and digestion buffer media centrifuged at 193× *g* for 3 min to collect the cells. Mouse tracheal epithelial cells (MTEC) were resuspended and placed in an air-liquid interface (ALI) Cell Nutrient Mix consisting of MTEC basic media (Hams F12:DMEM, 1:1) with growth supplements (Gibco-Thermo Fisher), counted, then seeded onto cell culture inserts with 1 × 10^5^ cells per insert and returned to the incubator. Once the cells reached confluency, the media were removed, and the cells were exposed to air until cilia formed (approximately 14 d).

### 2.2. Viability

Lactate dehydrogenase (LDH) release was measured in lung epithelial cells under all treatment conditions using a Lactate Dehydrogenase Activity Assay Kit (Sigma-Aldrich, Cat #MAK066). The assay was performed with cell culture media as a negative control and lysed cells as a positive control.

### 2.3. Alcohol Feeding Model

Mice receiving alcohol were given increasing concentrations of ethanol in water over a 2 wk period until the target concentration of 20% was reached. Mice in the alcohol group were given 5% alcohol (*w*/*v*) in a manner similar to that previously described [20]. Mice in the matched control group were given water from the same source without ethanol. All durations of alcohol exposure indicated in the remaining text refer to the time spent on the final 20% alcohol concentration. To ensure adequate intake of alcohol, blood alcohol concentrations (BACs) were measured using a kit by Pointe Scientific (Horiba, Canton Township, MI, USA). We detected a range of BAC in our mice of 20–50 mM, similar to that measured by others using this strain of mice [21]. All animal experiments were evaluated and approved by the University of Nebraska Medical Center Institutional Animal Care and Use Committee (Protocol #22-070-11-FC).

### 2.4. Particle Motility Assay

The movement of particles in mouse trachea was assayed using fluorescent infrared 500 nm latex microspheres labeled with Alexa Fluor 594 dye (Thermo Fisher, Waltham, MA, USA) detected for up to 20 min using the IVIS Lumina K Series III in vivo imaging system (Revvity, Waltham, MA, USA), and the movement of particles analyzed as a change in radiance Flux from carina to larynx over time. Trachea were excised from control and alcohol-fed mice after euthanasia, cleaned of connective tissue, and the lumen gently washed twice with 100 µL sterile PBS. All mouse trachea were approximately 6 mm in length, 1.5 mm in diameter, and contained 13–15 cartilage rings each. After a 1 h equilibration time in M-199 media at 37 °C and 5% CO_2_, each whole trachea was positioned onto a slide and placed into a 100 mm culture dish. Using a micropipette, 0.25 µL of infrared beads (500 nm) was added perpendicularly to the carina edge of the trachea and measured at multiple time points by IVIS at a heated-stage temperature of 37 °C for 12 min inside the covered culture dish. The total fluorescence flux (ρ/s) was quantified by drawing a region of interest (ROI) around the entire excised tissue. The initial total flux was similar between all tracheas and at all time points. The change in flux over time (Dflux) was determined by measuring the ROI of the right half (larynx side) of the trachea.

### 2.5. Zinpro Zinc LG Source and Preparation

**Figure 1:**
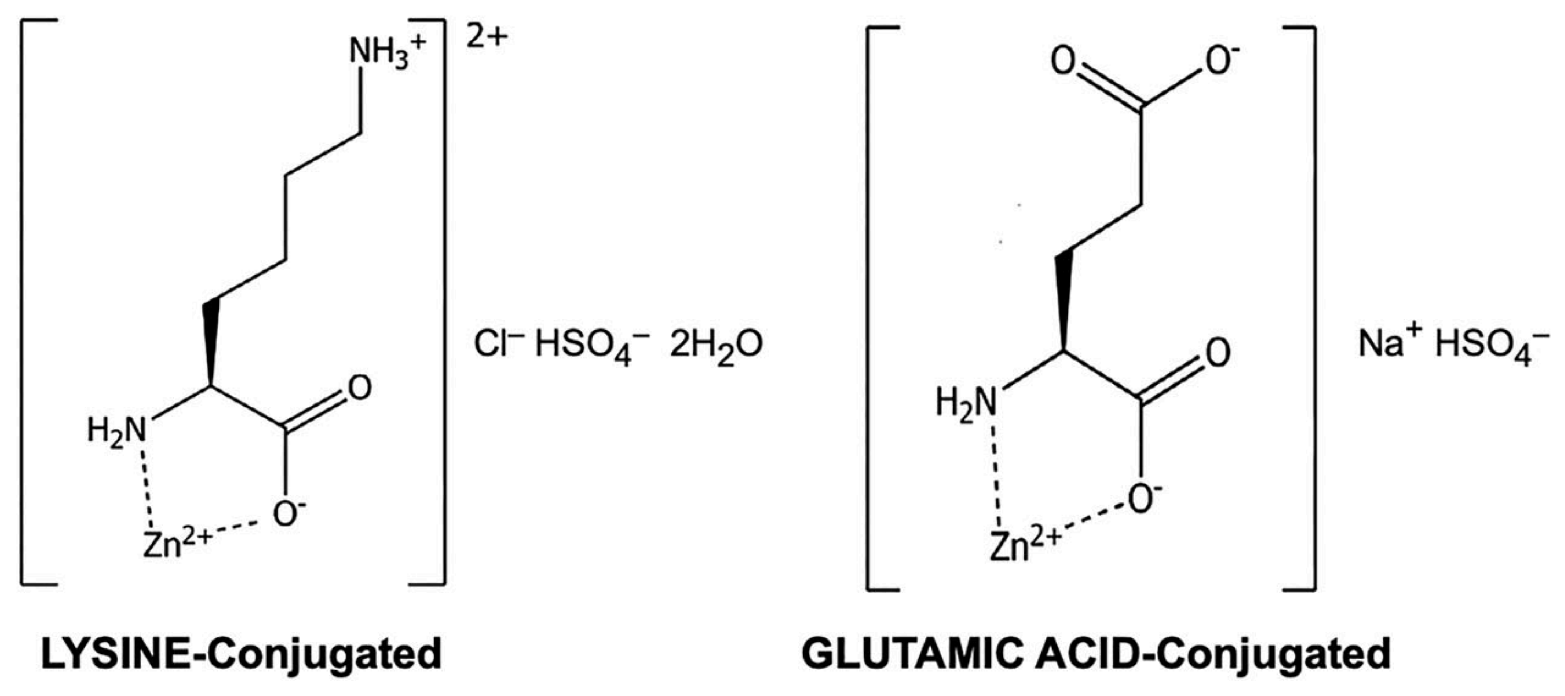
Zinpro Zinc LG is an amino acid chelate consisting of equal parts lysine and glutamic acid conjugated to zinc for enhanced cellular transport.

Zinpro Zinc LG is a form of zinc that is conjugated to the amino acids lysine and glutamic acid (Figure 1) for the purpose of enhancing cellular uptake and bioavailability in vivo. The Zinpro Corporation (Minneapolis, MN, USA) kindly provided the reagent used in this study, which was in the form of Glutamic Acid:Lysine (1:1). We previously determined the bioeffective concentrations (50 ng/mL to 1 μg/mL) of ZinPro Zinc LG on CBF in terms of time and concentration [6]. At very high concentrations of ZinPro Zinc LG (0.1–1 mg/mL), ciliated cell detachment and cytotoxicity were observed. Cilia slowing was prevented when ciliated cells were pretreated for 1 h with ZinPro Zinc LG at concentrations of 0.5 and 1.0 μg/mL, whereas ZinPro Zinc LG concentrations below 0.5 μg/mL did not reverse cilia slowing.

### 2.6. Cilia Beat Frequency Assay

A detailed characterization of the Sisson-Ammons Video Analysis (SAVA) system can be found in [22]. SAVA uses whole-field analysis to analyze cilia beat frequency (CBF) while eliminating operator bias due to selection of areas containing aberrantly fast- or slow-moving cilia. SAVA was used to detect regions of ciliated wild-type MTEC that were pretreated for 1 h with or without Zinpro Zinc LG. Following pretreatment, 50 mM ethanol was added to the cell culture media and incubated at 37 °C at 5% CO_2_ for 24 h prior to cell treatment for 1 h with 1 nM procaterol, 1 µM isoproterenol, or 10 µM 8Br-cAMP (Sigma-Aldrich). Changes in CBF and total number of motile points were measured at multiple time points over 24 h.

### 2.7. Kinase Activity Assay

BEC and MTEC at 80% confluency were pretreated for 1 h with or without Zinpro Zinc LG. Following pretreatment, 50 mM ethanol was added to the cell culture media and incubated at 37 °C at 5% CO_2_ for 24 h prior to cell treatment for 1 h with 1 nM procaterol, 1 µM isoproterenol, or 10 µM 8Br-cAMP. The cells were flash frozen with liquid N_2_ in 250 µL of a cell lysis buffer containing 35 mM 1M Tris-HCl (pH 7.4), 0.4 mM EGTA, 10 mM MgCl_2_, 10 mM phenylmethylsulfonyl fluoride, and a 1:100 dilution of Sigma Protease Inhibitor Cocktail (SPIC, Sigma-Aldrich). The cells were scraped, transferred into microfuge tubes, and dissociated by sonication for 5 s each. Catalytic transfer of phosphate from radiolabeled ^32^P-ATP onto specific substrate peptides was used to determine enzyme activity. Direct measurement of kinase activity was assayed for the cyclic AMP-dependent protein kinase (PKA) [23], cyclic GMP-dependent protein kinase (PKG) [24], and protein kinase C epsilon (PKCε) [25] as previously reported. For cell-free in vitro enzyme inhibition assays, Zinpro Zinc LG or amino acids (lysine/glutamine; 1:1) were added at various concentrations to purified PKA holoenzyme [26] or PKG [27], as well as commercially sourced catalytic subunit of PKA (Promega, Madison, WI, USA) and PKCε (SignalChem, Richmond, BC, USA), and enzyme activity assayed. Negative controls consisted of the absence of enzyme or substrate.

### 2.8. Measurement of Tissue Zinc Content

Whole lung from mice was collected and flash frozen until assayed. Lung samples were thawed and dried using a vacuum oven, then digested in 1 mL Nitric Acid:Perchloric Acid (1:2) for 2 h @ 80 °C. Zinc measurements were analyzed using a PinAAcle 500 Atomic Absorption Spectrometer (PerkinElmer, Shelton, CT, USA). Results were standardized by total weight (g) assayed.

### 2.9. Phosphatase Assay

Mouse tracheal epithelial homogenate protein concentrations were measured by the Bradford assay, and each sample was normalized to equal concentration. Samples were diluted 1:4 in cell lysis buffer and added to duplicate wells of a 96-well plate. Phosphothreonine-peptide (5.0 µL of a 1.0 mM solution of K-R-pT-I-R-R; Cat #12-219; Millipore, Burlington, MA, USA) was then added to each well to begin the reaction. The reaction was allowed to incubate at room temperature for 5 min. The reaction was then stopped with 100 µL of malachite green phosphate detection solution and allowed 15 min for color development. Using a microplate spectrophotometer (Aligent BioTek Epoch, Santa Clara, CA, USA), absorbance was read at 620 nm. Phosphate release was determined by a standard curve generated from the Ser/Thr Phosphatase Assay Kit (Cat #17-127, Millipore) supplied with a phosphate standard solution (Cat #20-103). Phosphatase activity was expressed as picomoles phosphate per minute per microgram of total protein.

### 2.10. Materials

All materials not specifically stated were obtained from Sigma-Aldrich.

### 2.11. Statistical Analysis

For in vitro human and mouse cell experiments, 3–5 separate biological replicates of independent experiments were performed, consisting of triplicate technical replicates for each separate experiment. For the mouse trachea ex vivo data, 12 biological subjects were used for each treatment condition. For the zinc tissue assays, 10 biological replicates were used. Determination of data normality was conducted by the D’Agostino and Pearson test (alpha = 0.05), and the residuals were determined to be normally distributed. Data are presented as standard deviation (SD) with scatter plots for individual mice depicted for each treatment. Student’s *t*-test was used to assess statistical differences between two groups; one-way analysis of variance (ANOVA) was used to assess statistical differences among 3 or more experimental groups with Tukey’s post hoc correction for multiple comparisons between any two groups. Statistical significance was set at a threshold *p*-value of <0.05. Statistical analyses were performed using GraphPad Prism 10 (San Diego, CA, USA) software.

## 3. Results

### 3.1. Alcohol Consumption Slows Mucociliary Clearance

**Figure 2:**
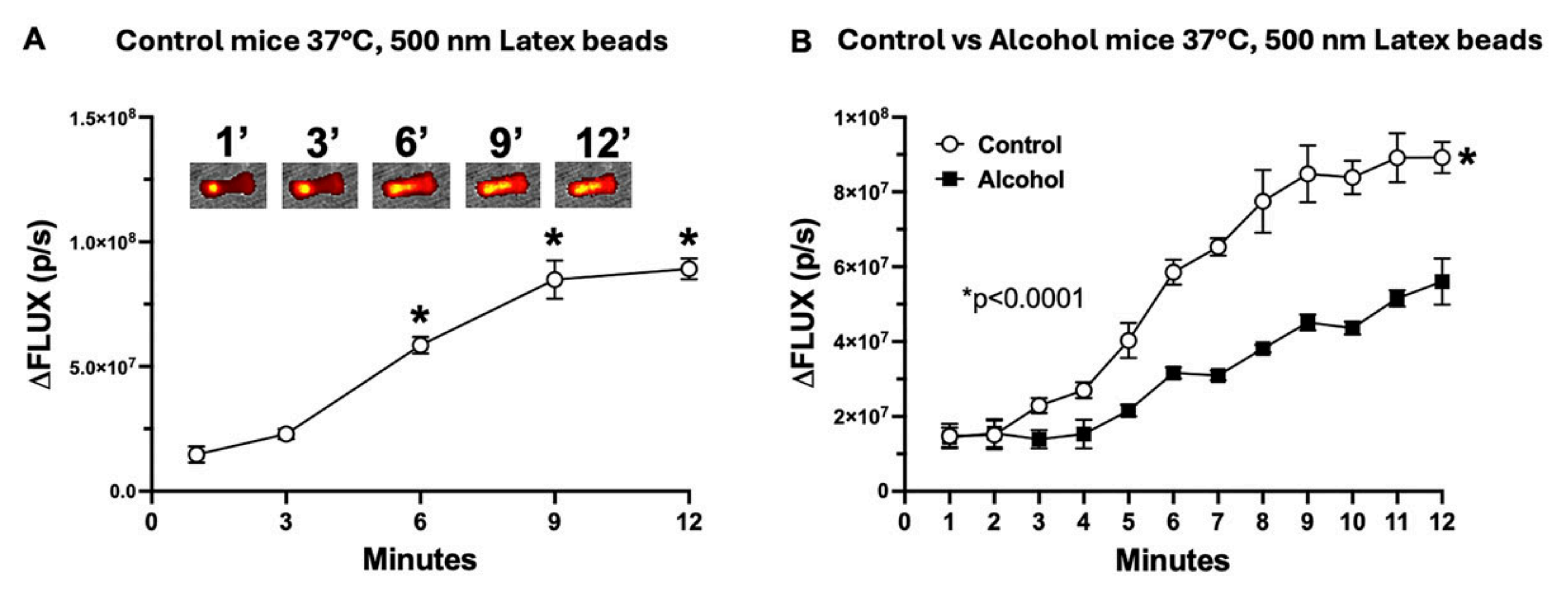
Alcohol feeding in mice slows mucociliary clearance in an ex vivo tracheal bead movement assay. Particle movement across the trachea was measured as a change in radiance flux across distal regions of interest (ROI). (**A**) Representative images of infrared-labeled 500 nm latex bead movement over 1–12 min from bronchial bifurcation to cricoid in excised control mice; (**B**) Particle movement in Control water-fed mice vs. 8-week 20% alcohol-fed mice. (* *p* < 0.001 6–12 min vs. 1–3 min; *p* < 0.0001 Control vs. Alcohol, two-way ANOVA, *n* = 12 mice per treatment).

AICD has been established in both human and animal cell models, and the functional consequences of alcohol on mechanical clearance in mice are typically demonstrated through enhanced lung infection models. We developed a mouse tracheal particle transport model to link cilia beating and functional clearance. Using infrared-labeled latex beads of the optimum size and surface chemistry to stimulate cilia motility [28], stimulated movement of beads from the carina to the cricoid of the excised trachea could be observed by 12 min as determined by maximum change in radiant photon flux from the distal to the proximal end (Figure 2A). However, a significant (*p* < 0.0001) decrease in particle movement was observed in alcohol-fed mice vs. control mice (Figure 2B). These data demonstrate a functional consequence of AICD in mice.

### 3.2. Inorganic Zinc Salts Have No Effect on AICD

**Figure 3:**
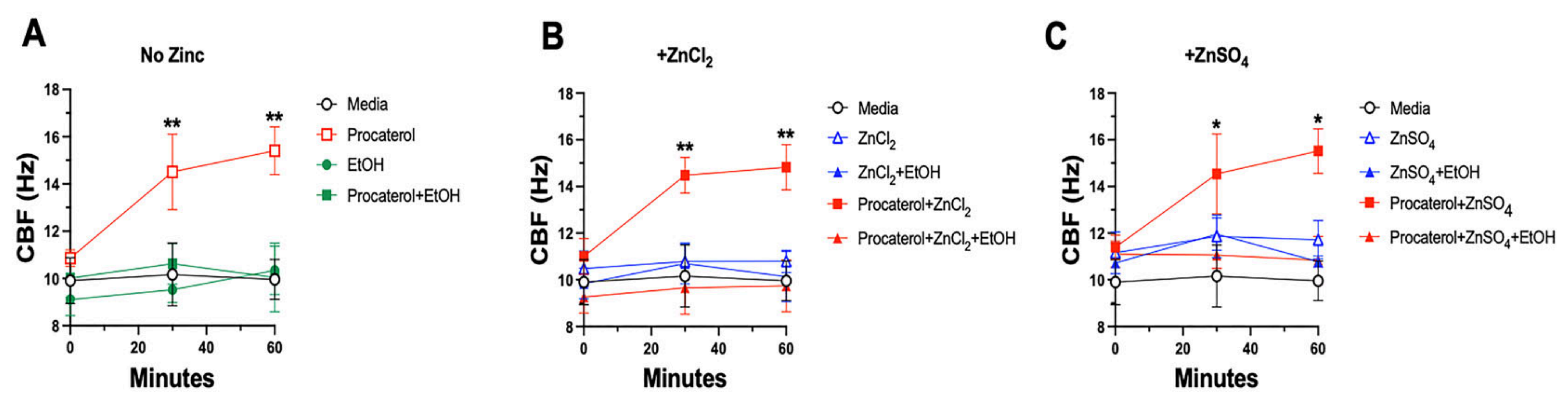
Alcohol-induced desensitization of stimulated ciliary beat frequency (CBF) in cultured mouse tracheal epithelial cells (MTEC) is unaffected by pretreatment with inorganic salts of zinc. (**A**) CBF in MTEC cultured on an air-liquid interface and treated with or without procaterol (10 nM) for 1 h in the presence or absence of 24 h pretreatment with 50 mM alcohol (EtOH). Zinc (10 µg/mL) in the form of ZnCl_2_ (**B**) or ZnSO_4_ (**C**) has no effect on baseline, procaterol-stimulated, or alcohol-desensitized cilia. (* *p* < 0.03; ** *p* < 0.006; one-way ANOVA, *n* = 5 independent experiments consisting of 3 technical replicates each).

Zinc chloride is reported to stimulate CBF in mouse nasal ciliated cells [17]. We investigated whether zinc treatment of ciliated MTEC could prevent AICD. As previously reported [29], a 1 h exposure to beta-agonist (10 nM procaterol) stimulated increases in CBF, which are prevented when the MTEC are pre-treated with 50 mM alcohol for 24 h (Figure 3A). Pretreatment of MTEC for 1 h prior to overnight alcohol with zinc (10 µg/mL) in the form of ZnCl_2_ (Figure 3B) or ZnSO_4_ (Figure 3C) had no effect on baseline, procaterol-stimulated, or alcohol-desensitized cilia. These results revealed no significant effects of either zinc chloride or sulfate salts on baseline CBF or AICD in tracheal cilia.

### 3.3. Zinpro Zinc LG Restores cAMP-Stimulated Cilia After Alcohol Exposure

**Figure 4:**
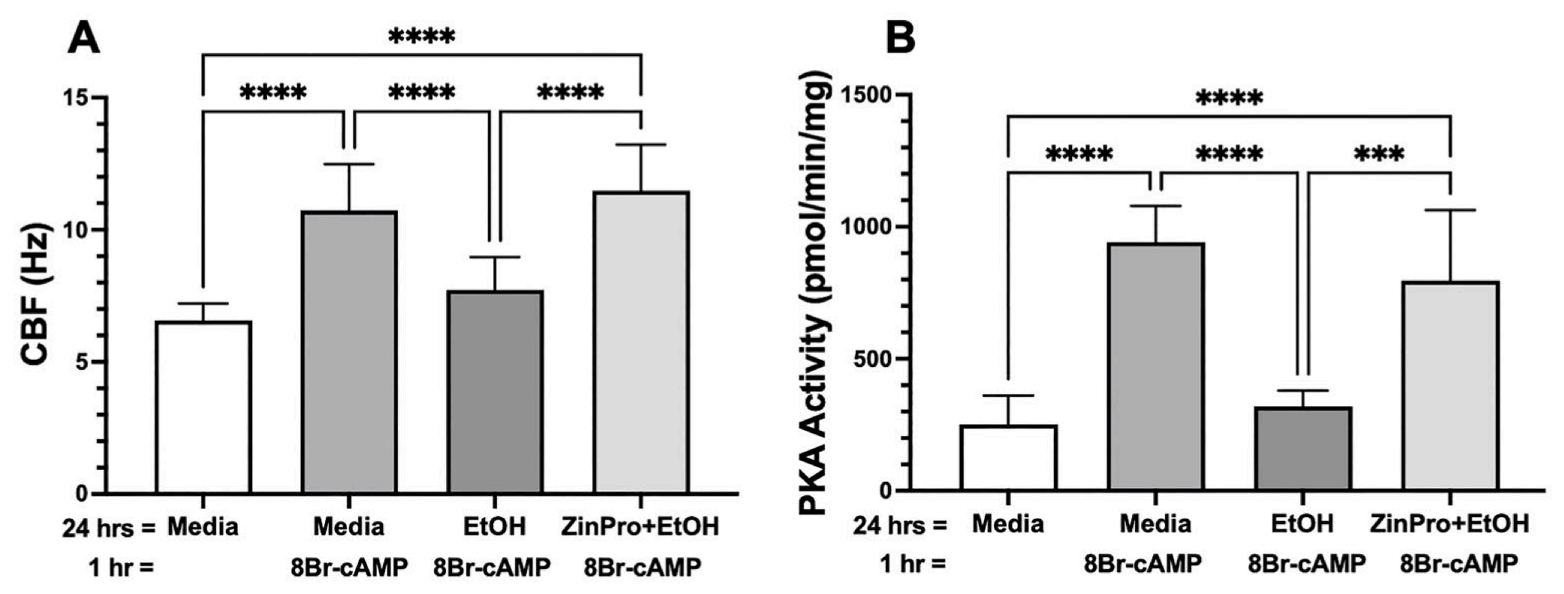
Zinpro Zinc LG prevents alcohol-mediated desensitization of (**A**) cilia or (**B**) cyclic AMP-dependent protein kinase (PKA) in primary mouse tracheal epithelial cells (MTEC) grown on air-liquid interface. MTEC were treated with or without 1 µg/mL Zinpro Zinc LG for 1 h followed by control media or 50 mM alcohol (EtOH) for 24 h and then exposed to 10 µM 8Br-cAMP for 1 h. Alcohol-induced ciliary dysfunction (AICD) could be blocked in mouse ciliated cells pretreated with Zinpro Zinc LG before alcohol exposure. (*** *p* < 0.01; **** *p* < 0.001; one-way ANOVA, *n* = 4 independent experiments consisting of 3 technical replicates each).

Recently, we observed that a cell permeability-enhanced equimolar combination of amino acid-conjugated zinc, Zinpro Zinc LG, was effective at protecting against organic dust-mediated cilia slowing [6]. We then sought to investigate whether Zinpro Zinc LG could be protective against the alcohol-mediated desensitization of cilia stimulation. MTECs were treated with or without 50 mM alcohol for 24 h, followed by a 1 h cilia stimulation with a cell-permeable analog of cAMP, 8-bromo-cAMP, and again revealed AICD as alcohol prevented both the cAMP stimulation of CBF (Figure 4A) and activation of the ciliostimulatory kinase, PKA (Figure 4B). However, a 1 h pretreatment with 10 µg/mL Zinpro Zinc LG prior to alcohol exposure prevented the loss of cAMP-stimulated PKA and CBF. Zinpro Zinc LG alone had no effect on motility or PKA activity. These data indicate that uptake of Zinpro Zinc LG occurs in MTECs and that the action of intracellular zinc on AICD takes place upstream of PKA.

### 3.4. Zinpro Zinc LG Prevents Alcohol-Induced Desensitization of PKA in Human Airway Epithelial Cells

**Figure 5:**
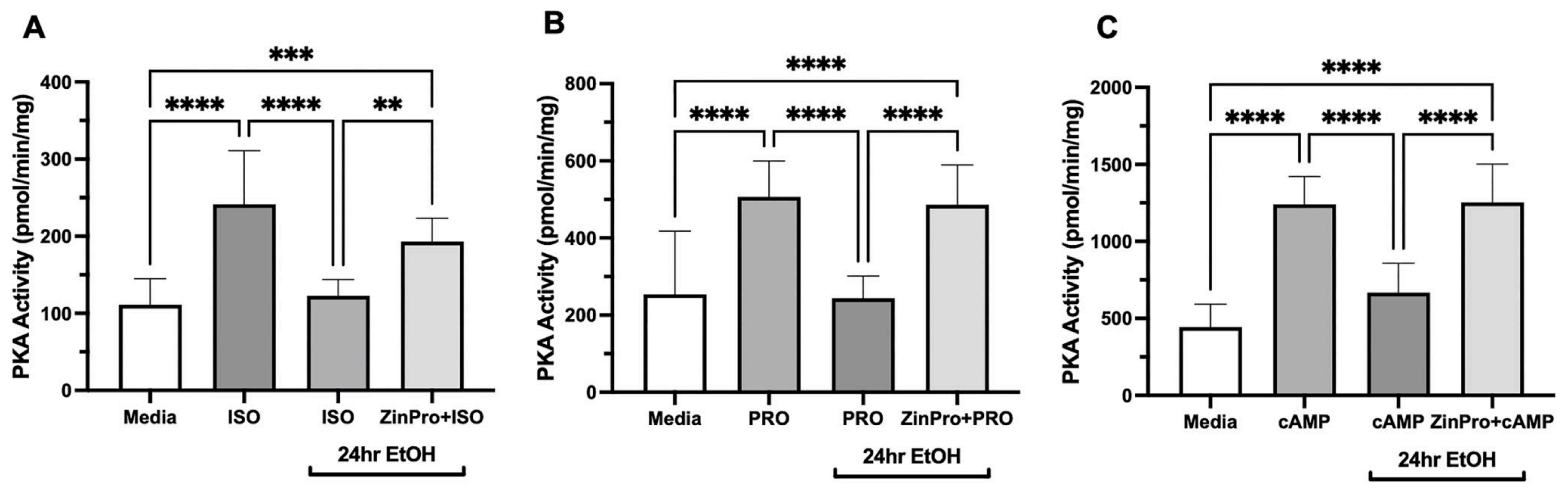
Pretreatment with Zinpro Zinc LG prevents alcohol-mediated desensitization of PKA in a human bronchial epithelial cell line, BEAS-2B. Zinpro Zinc LG (1 µg/mL) prevented the alcohol desensitization of PKA activation in response to (**A**) 1 µM of the short-acting beta agonist isoproterenol (ISO), (**B**) 10 nM of the long-acting beta agonist procaterol (PRO), or (**C**) 10 µM of the cell-permeable PKA agonist 8Br-cAMP (cAMP) in BEAS-2B cells. (** *p* < 0.001;*** *p* < 0.0002; **** *p* < 0.0001; one-way ANOVA, *n* = 4 independent experiments consisting of 3 technical replicates each).

To further support the protective effects of Zinpro Zinc LG on AICD, we repeated the treatment groups using a human bronchial epithelial cell line (BEAS-2B) using both a short-acting beta-agonist, isoproterenol (1 µM; Figure 5A), and a long-acting beta-agonist, procaterol (10 nM; Figure 5B). As with MTEC, 10 µg/mL pretreatment with Zinpro Zinc LG restored the ability of both beta-agonists to stimulate PKA after alcohol desensitization. Similarly, direct activation of PKA by 8Br-cAMP occurred after 24 h alcohol treatment in BEAS-2B when the cells were first pretreated with Zinpro Zinc LG (Figure 5C). As with MTEC, these data suggest zinc protection from AICD downstream of membrane-bound beta-receptor.

### 3.5. PKG Activity Is Not the Target of Zinpro Zinc LG Protective Effect on AICD in Response to Beta-Agonists

**Figure 6:**
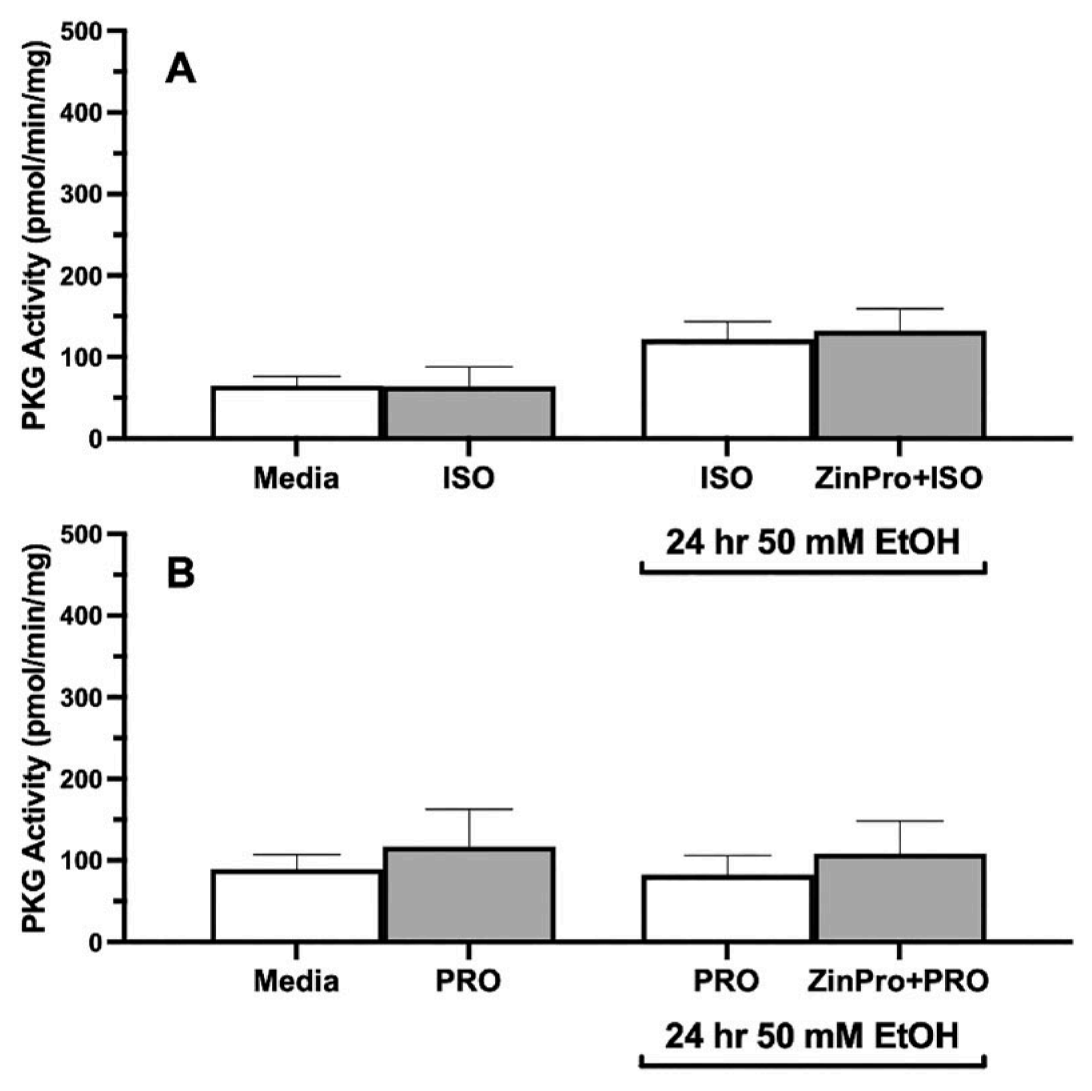
Zinpro Zinc LG does not alter cyclic GMP-dependent protein kinase (PKG) activity in BEAS-2B. Cells were pretreated for 1 h with 1 µg/mL Zinpro Zinc LG followed by media or 50 mM alcohol (EtOH) for 24 h and then exposed to PKA stimulatory conditions of (**A**) 1 µM isoproterenol (ISO) or (**B**) 10 nM procaterol (PRO) for 1 h. (*p* > 0.94; one-way ANOVA, *n* = 2 independent experiments consisting of 3 technical replicates each).

Previous reports describe the activation of PKA by zinc-stimulated cGMP increases in monocytes [30]. The cGMP pathway is known to stimulate CBF [24], and because cyclic nucleotide cross-activation of PKG by cAMP is possible [31], we examined the potential for cellular PKG activity in the presence or absence of Zinpro Zinc LG under AICD conditions of beta-agonist stimulation. No activation of PKG was observed under ciliostimulatory conditions of 10 µM isoproterenol (Figure 6A) or 1 nM procaterol (Figure 6B). Most noteworthy, the addition of 10 µg/mL Zinpro Zinc LG alone or prior to 24 h of alcohol resulted in no measurable changes in PKG activity.

### 3.6. In Vitro Purified PKCε, but Not PKA or PKG, Is Inhibited by Zinpro Zinc LG

**Figure 7:**
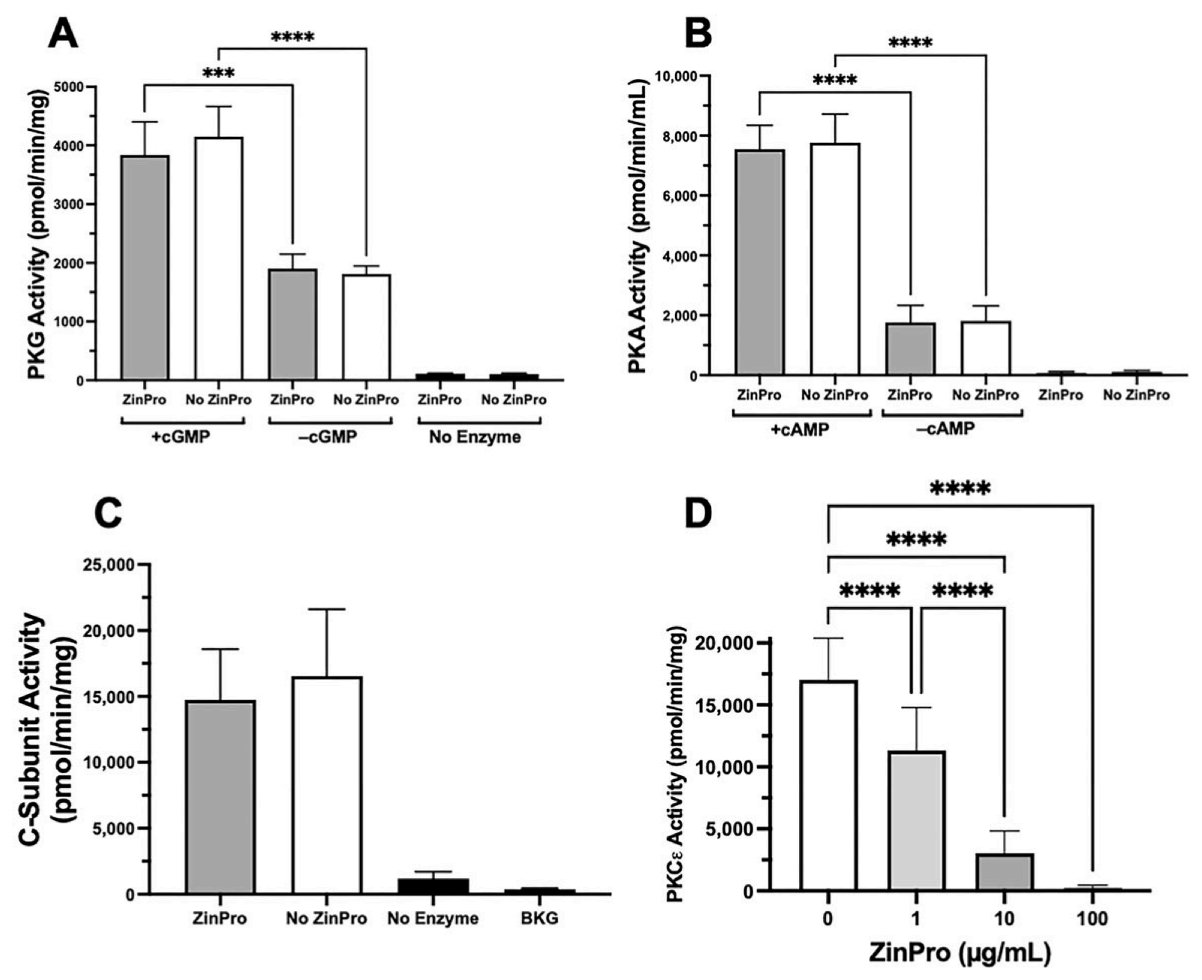
Using purified enzyme protein, Zinpro Zinc LG does not directly inhibit the in vitro enzyme activity of PKG, PKA, or the catalytic subunit (C-subunit) of PKG, but does inhibit PKCε in a concentration-dependent manner. Purified PKG (**A**), PKA holoenzyme (**B**), or C-subunit of PKA (**C**) were assayed for phosphorylation of their specific peptide substrates in the presence or absence of 10 µg/mL Zinpro Zinc LG. Purified PKCε was inhibited by 1–10 µg/mL of Zinpro Zinc LG (**D**). (*** *p* < 0.001; **** *p* < 0.0001; one-way ANOVA, *n* = 3 independent experiments consisting of 3 technical replicates each).

Because many enzymes contain zinc-binding sites that inhibit their activity [32], we measured direct enzyme activities of purified kinases that regulate CBF in airway epithelial cells. As with intact cell-associated PKG, we observed no change in baseline or cGMP-stimulated purified PKG activity in the presence of 10 µg/mL Zinpro Zinc LG (Figure 7A). Similarly, Zinpro Zinc LG treatment resulted in no changes in the in vitro enzyme activity of either purified PKA holoenzyme (Figure 7B) or Catalytic Subunit of PKA (Figure 7C). Most striking, unlike the cyclic nucleotide ciliostimulatory kinases, Zinpro Zinc LG inhibits in vitro purified PKCε activity in a dose-dependent manner (Figure 7D).

### 3.7. Alcohol Feeding in Mice Does Not Decrease Total Zinc Content in Lung

**Figure 8:**
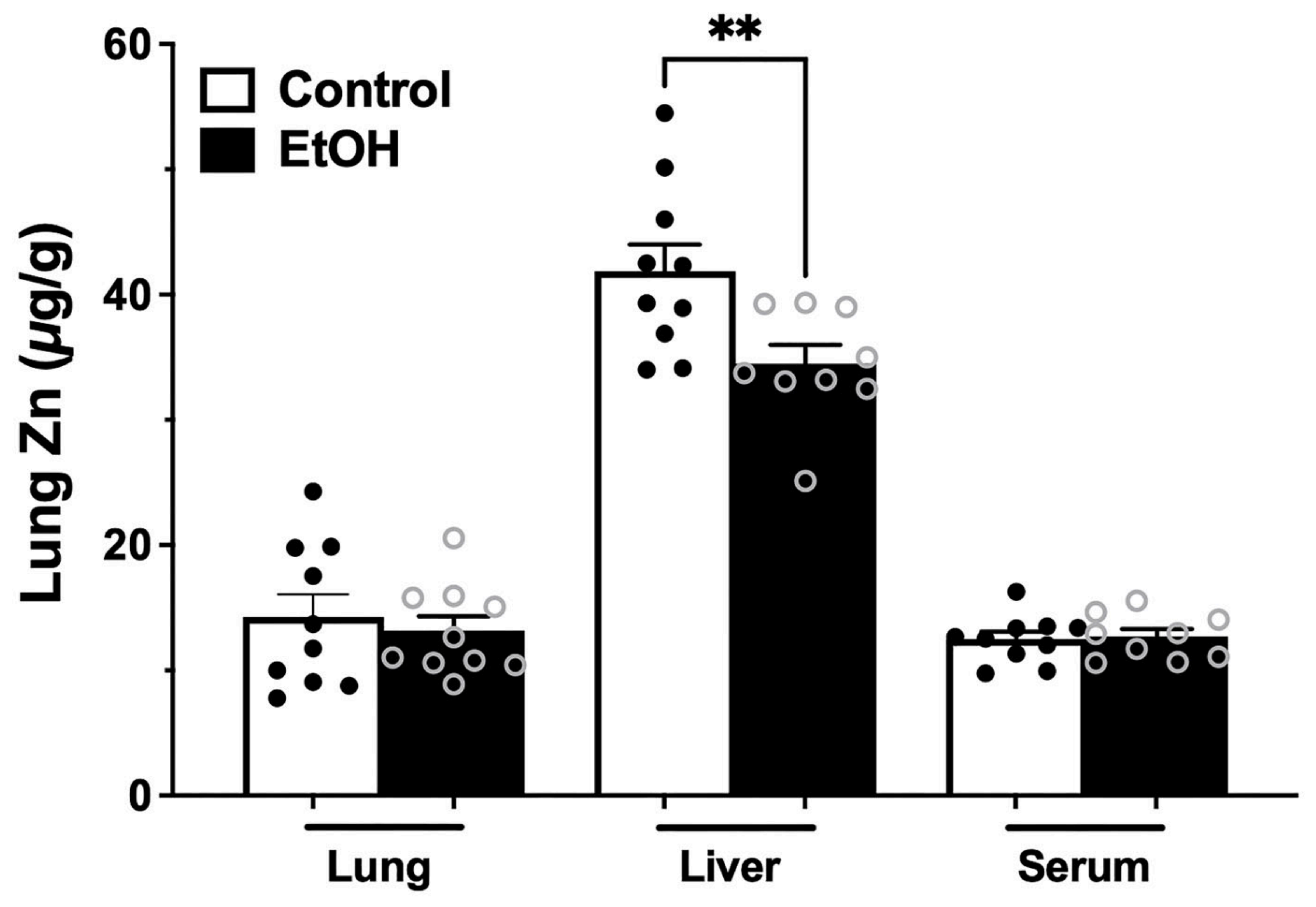
Whole tissue Zinc levels in lung, liver, and serum of mice fed a control diet vs. alcohol-fed mice measured by atomic absorption spectroscopy. Alcohol fed mice have only a small, but not significant, decrease in total zinc content in the lung. In contrast, alcohol reduces liver zinc content. (** *p* < 0.03; one-way ANOVA, *n* = 10 mouse biological replicates).

People with alcohol use disorders often have zinc deficiency, particularly with regard to liver [33]. We examined whole tissue zinc levels in the lung to determine if murine AICD was coincident with decreased zinc. No significant differences were detected in lung or serum from control diet mice vs. alcohol-fed mice (Figure 8). However, consistent with our previous work [34] liver zinc levels were significantly reduced in the alcohol-fed mice.

### 3.8. Zinpro Zinc LG Inhibits Alcohol-Stimulated Protein Phosphatase 1

**Figure 9:**
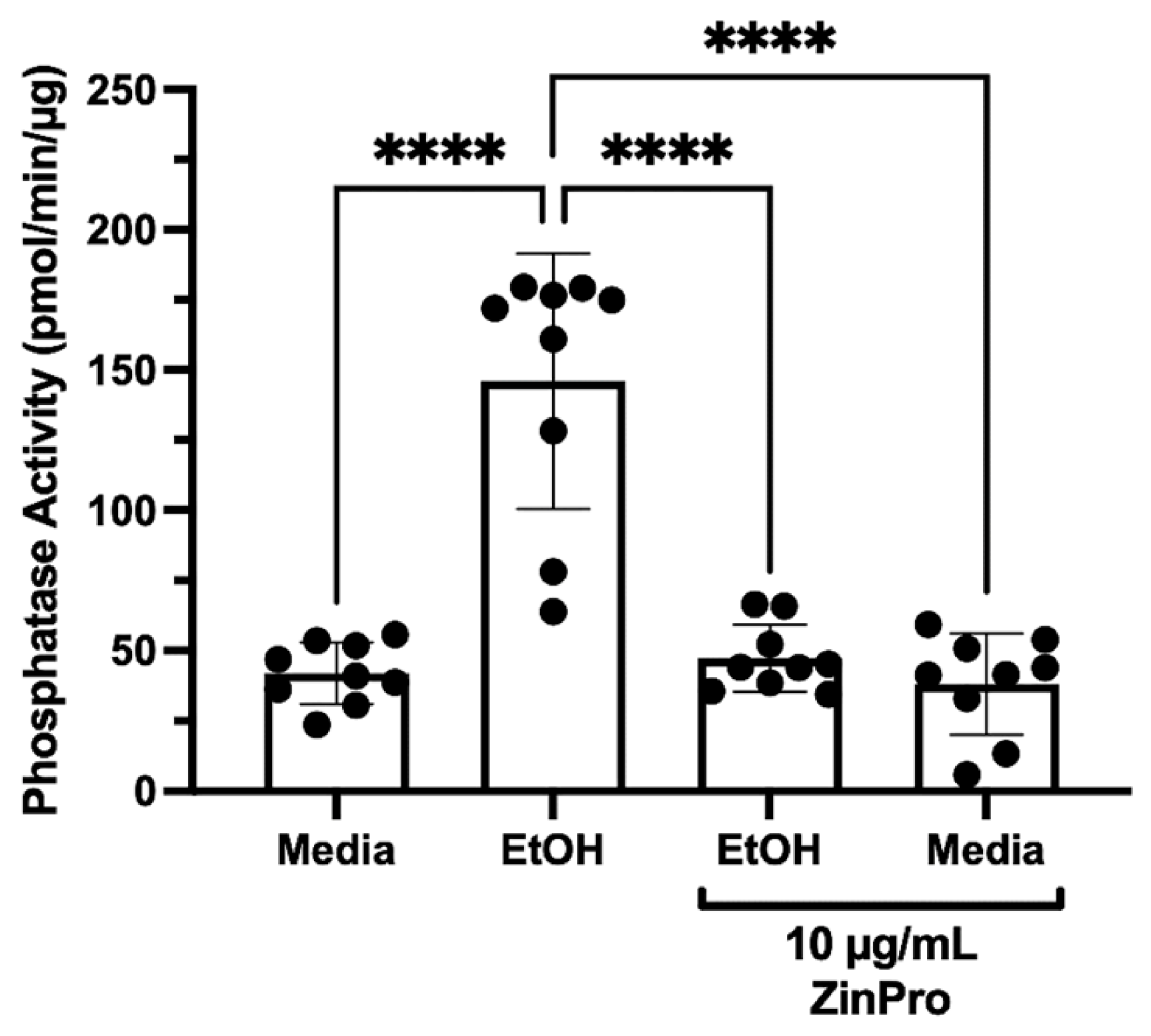
Zinpro Zinc LG inhibits alcohol stimulated protein phosphatase 1 (PP1) activity in mouse tracheal epithelial cells. Epithelia isolated from mouse trachea in vitro were treated with or without 50 mM ethanol (EtOH) for 24 h in the presence or absence of 10 µg/mL Zinpro Zinc LG. (**** *p* < 0.0001; one-way ANOVA, *n* = 3 independent experiments consisting of 3 technical replicates each).

Zinc can inhibit protein phosphatase 1 (PP1; [35]), and the AICD signal transduction pathway involves alcohol-stimulated increases in PP1 [7]. Because of this, we measured mouse tracheal PP1 in the presence or absence of alcohol and Zinpro Zinc LG. Alcohol (50 mM for 24 h) significantly (*p* < 0.0001) stimulated PP1 activity compared to control media (Figure 9). However, pretreatment with 10 µg/mL Zinpro Zinc LG for 1 h prior to alcohol exposure prevented increased activity in mouse tracheal epithelium. These data demonstrate that the beneficial impact of Zinpro Zinc LG against AICD is a consequence of inhibition of PP1 phosphatase activity.

## 4. Discussion

**Figure 10:**
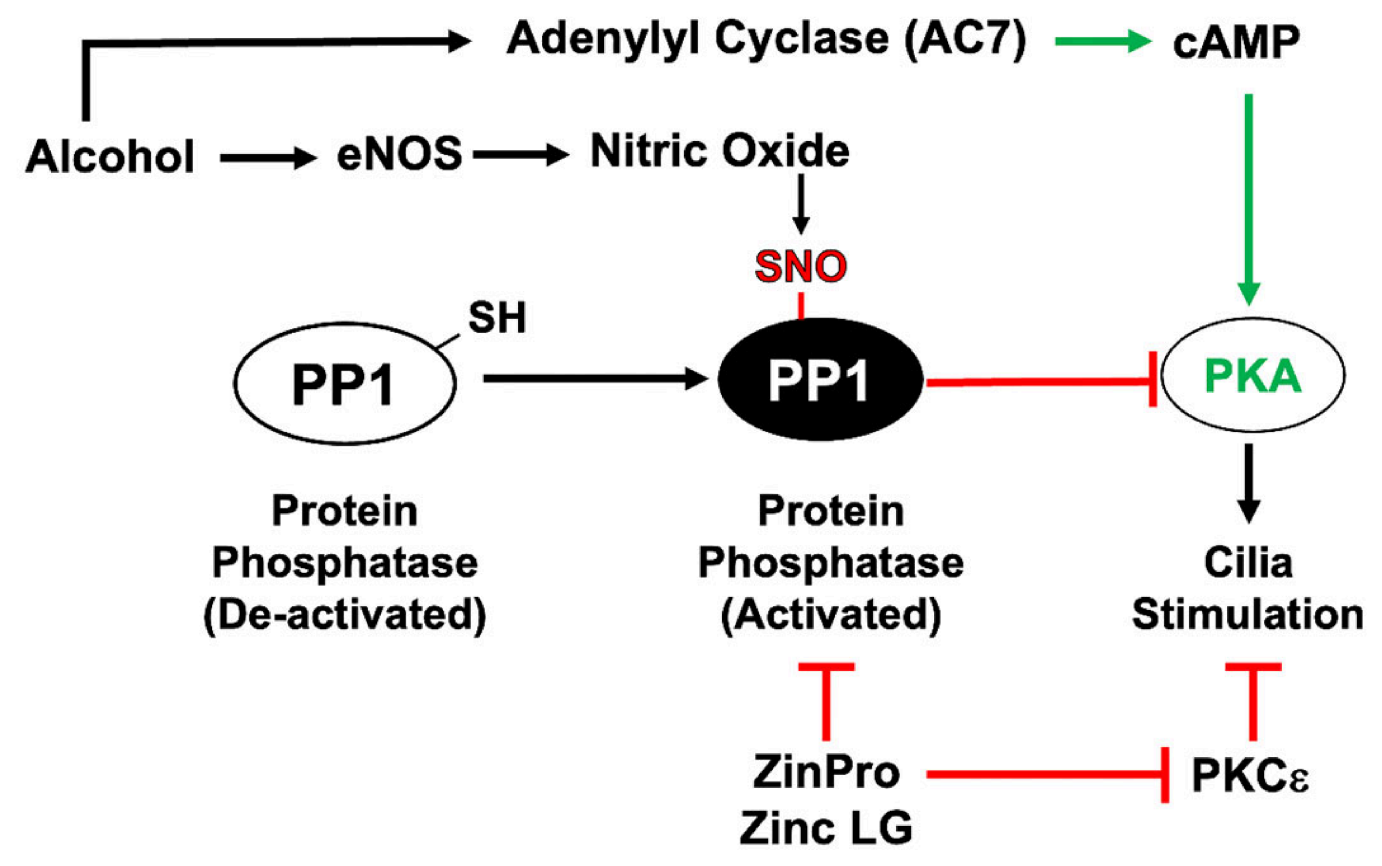
Summary diagram of AICD action and potential Zinpro Zinc LG targets. Alcohol can rapidly stimulate short-term cilia motility through an alcohol-activated adenylyl cyclase (AC7), but longer-term alcohol exposure desensitizes ciliary protein kinase A (PKA) from further stimulated beating increases via the alcohol stimulation of a nitric oxide synthase (eNOS), leading to the S-nitrosylation (SNO) of protein phosphatase 1 (PP1). Zinpro Zinc LG inhibits PP1 as well as protein kinase C epsilon (PKCε), a kinase capable of slowing cilia. Green arrows indicate activation pathway and red tee-heads indicate inhibition of pathway.

Alcohol negatively impacts numerous innate and adaptive defense mechanisms in the lung, leading to increased susceptibility to infections and pneumonia. One such mechanism is defined as alcohol-induced ciliary dysfunction [29], whereby alcohol blunts the stimulation of increased cilia motility by inhaled particles and pathogens, resulting in decreased mechanical clearance. Previous studies [7] have identified that AICD occurs when the normal rapid and brief stimulation of cilia by alcohol via the stimulation of an alcohol-sensitive adenylyl cyclase (AC7) subsides, while continued alcohol exposure results in the S-nitrosylation of PP1, leading to the desensitization of PKA activation (Figure 10). Our findings demonstrate that cellular zinc uptake using Zinpro Zinc LG, a zinc conjugated with lysine and glutamic acid for enhanced cellular transport via amino acid transporters [36,37], can inhibit alcohol-activated PP1 and prevent AICD in a ciliated cell.

Several lines of investigation establish the plausibility of PP1 inhibition by zinc. Zinc is known to bind to numerous enzyme active sites, including protein phosphatases, resulting in the inhibition of enzyme function [32]. Nanoparticles conjugated with zinc oxide for enhanced delivery to a neuroblastoma cell line resulted in a significant decrease in both PP1 protein levels and enzyme activity [35]. In our own previous studies, we identified that inhibiting PP1 by blocking S-nitrosylation of the enzyme, or by generating a mutant inactive PP1 cell line, resulted in the elimination of AICD [38]. PP1 inhibitors function to produce the same prevention of AICD [39]. We now show that the organic zinc formulation Zinpro Zinc LG is superior to conventional inorganic zinc salts through PP1 inhibition, thereby suppressing AICD and, in doing so, indirectly impacting the ciliostimulatory action of PKA. Unlike sperm or bone-marrow mesenchymal cells [40], our data show no direct activation of PKA (or PKG) by Zinpro Zinc LG in ciliated airway cells.

Rather than the suppression of cilia-stimulated increases in beating seen in AICD, several inhaled substances, such as cigarette smoke and organic dusts, result in cilia slowing below that of baseline beating. These agents share a common pathway of PKCε activation [41]. Hazardous air-quality occupational environments, such as in swine confinement feeding facilities, are responsible for inflammatory-based lung disease. Studies have shown that organic dust extracts from swine barns significantly decreased CBF after 24 h exposure via the activation of PKCε [42]. This mechanism was prevented when ciliated cells were pretreated with Zinpro Zinc LG, as our findings show a direct inhibitory action of zinc on PKCε [6]. In support of this finding, others have demonstrated zinc inhibition of phosphorylation of the novel PKC isoform, PKCδ [43]. The threshold amount of reactive aldehydes generated by the combination of alcohol and cigarette smoke exposure results in PKCε activation, cilia slowing, and cilia loss [44]. This represents an important additional mechanism of Zinpro Zinc LG in those with AUD, as the vast majority of individuals with AUD also smoke cigarettes.

A key element to our findings is the importance of zinc transport and the innovative nature of Zinpro Zinc LG in enhancing cellular uptake. While an early study of nasal cilia reported ZnCl_2_-stimulation of CBF [17], this effect only occurred in the presence of high concentrations of calcium as well. In our studies, we observed no stimulatory effect on airway epithelial CBF with the inorganic zinc salts, ZnCl_2_ or ZnSO_4_. In fact, very high concentrations (200 µM) of ZnCl_2_ inhibited CBF in mouse tracheal ciliated epithelium [45]. Likewise, we observed detachment of ciliated cells and cytotoxicity with high concentrations of Zinpro Zinc LG (1 mg/mL; [6]). In contrast, concentrations of Zinpro Zinc LG that are physiologically relevant to blood zinc levels (10 µg/mL) prevented AICD in airway epithelial cells while producing no stimulatory or inhibitory effects on CBF in and of itself. This finding points to effective uptake of zinc in the form of the amino acid conjugate Zinpro Zinc LG. Increased zinc uptake from Zinpro Zinc LG and enhanced barrier function was demonstrated in the epithelial cell line Caco-2 [46].

An interesting observation in our study is that alcohol feeding did not significantly lower total tissue zinc content in the lung. As we have previously published [47,48], whole-tissue zinc levels in humans and mice are often misleading and do not reflect concentrations under physiological and/or pathologic conditions at the cellular level. Mouse tissue zinc levels typically appear normal even under pathologic conditions unless mice are subject to severely restricted zinc intake in their diet for prolonged (weeks) periods of time. This is in large part due to the existence of two families of zinc transporters (14 ZIP importers and 10 ZnT exporters). The expression and function of each transporter is tissue-, cell-, and organelle-specific relative to maintaining cellular abundance and availability in the cytosol, as well as entry and exit from organelles. Their expression is also subject to change under stressful conditions. Our observation that zinc-specific changes in kinase activity within epithelial cells occurred despite what appear to be relatively normal lung tissue concentrations is consistent with our past observations and those of others [49,50].

While our initial findings reported here are novel in that they represent the first in vitro data implicating zinc as a potential agent to address AICD, there are several limitations to this study that must be addressed through future research before conclusions can be made with regard to Zinpro Zinc LG as a prophylactic or therapeutic agent. Comparisons with other commercial bioavailable zinc formulations were not performed, as this study only utilized Zinpro Zinc LG. In this study, we performed in vitro kinase activity assays to evaluate the effect of Zinpro Zinc LG on purified enzyme catalytic activity. Such data do not address the direct binding site of zinc to the target enzyme, which will require studies showing co-localization or immunoprecipitation of PP1-bound zinc in the intact cell. No conclusions can be made at this time about the multi-organ impact of zinc resolution of AICD. While numerous preclinical alcohol feeding animal models exist, our study uses only the Cook-Meadows ad libitum alcohol in drinking water model [20], which is shown to cause lung AICD. This model produces a blood alcohol content (BAC) in mice as high as 80 mM. Because approximately 20 mM BAC is a legal limit in most jurisdictions for the operation of a motor vehicle, we utilized 50 mM in our in vitro study to mirror the pathophysiologic levels observed in heavily intoxicated individuals. Mice on an individual basis consume variable amounts of alcohol, and we cannot rule out the interference of feeding volume differences on the experimental results, which must be presented in the aggregate. Lastly, no in vivo mouse supplementation experiments or human clinical trials were performed in the present study. Therefore, any proposed therapeutic potential of Zinpro Zinc LG remains speculative.

To address these limitations, future studies will need to clarify the specific binding domains and molecular sites of zinc to PP1/PKCε. Molecular docking or point-mutation verification experiments will be required to support the existing enzyme inhibition findings, as well as using molecular simulation and PP1 mutation cell lines to verify the key binding sites for Zinpro Zinc LG. Ongoing experiments will identify the optimal administration model for Zinpro Zinc LG in alcohol-fed mice to evaluate the restoration of normal tracheal particle clearance to establish whether oral administration of Zinpro Zinc LG reaches the airway epithelium at effective concentrations in the intact mouse. Likewise, future validation of these functional and mechanistic findings needs to be conducted using in vitro primary human ciliated cells using carefully characterized donors for AUD and smoking status, as a logical next step for clinical translation.

## 5. Conclusions

Zinc deficiency has long been associated with increased susceptibility and severity of respiratory infections. Zinc deficiency and lung infections have manifested with regard to COVID-19 [12] and pediatric respiratory infections [51]. A recent meta-analysis from 34 studies demonstrates that while zinc supplementation does not prevent the common cold, the duration and severity of colds are diminished by zinc [52]. Alcohol misuse has long been associated with increased risk for pneumonia, but less attention has been focused on the observation that people with alcohol use disorders have increased rates of zinc deficiency [33]. Our findings identify an in vitro cellular relationship between zinc, AICD, and ciliary function. From a translational perspective, future studies may represent a tractable, new, preventive treatment strategy to address the public health problem of alcohol and respiratory infections.

## Data Availability

The original contributions presented in this study are included in the article. Further inquiries can be directed to the corresponding author.

## Abbreviations

The following abbreviations are used in this manuscript:

AICD	Alcohol-induced ciliary dysfunction
ALI	Air-liquid interface
ANOVA	Analysis of variance
ATCC	American Type Culture Collection
AUD	Alcohol use disorder
BAC	Blood alcohol content
CBF	Cilia beat frequency
DMEM	Dulbecco’s Modified Eagle Media
FBS	Fetal bovine serum
LDH	Lactate dehydrogenase
MTEC	Mouse tracheal epithelial cells
PKA	cAMP-dependent protein kinase
PKCε	Protein kinase C epsilon
PKG	GMP-dependent protein kinase
PP1	Protein phosphatase 1
ROI	Region of interest
SAVA	Sisson-Ammons Video Analysis
SD	Standard deviation
SPIC	Sigma Protease Inhibitor Cocktail
ZIP	Zrt, Irt-like
ZnT	Zinc Transporter
